# Reward and punishment learning among people with a lifetime history of anxiety, depression, and substance use disorder

**DOI:** 10.3758/s13415-025-01331-y

**Published:** 2025-07-29

**Authors:** Jeremy M. Haynes, Holly Sullivan‑Toole, Nathaniel Haines, Thomas M. Olino

**Affiliations:** 1 Department of Psychology, Temple University, 1701 N 13Th St, Philadelphia, PA 19122, USA; 2 University of Minnesota, 2025 E River Parkway, Minneapolis, MN 55414, USA; 3 Bayesian Beginnings LLC, 1581 N High St, Columbus, OH 43201, USA; 4 Present Address: Department of Psychology, Georgia Southern University, 1332 Southern Dr, Statesboro, GA 30458, USA

**Keywords:** Iowa gambling task, Anxiety, Depression, Substance use disorder, Computational modeling, Hierarchical Bayesian analysis

## Abstract

Reward and punishment learning are critical across multiple clinical populations. The Iowa Gambling Task (IGT) is frequently used to assess these constructs and multiple forms of psychopathology are associated with IGT performance. However, it is not clear whether alterations in IGT performance are general to psychopathology or specific to different forms of psychopathology. Thus, we examined whether IGT performance was uniquely predicted by anxiety, depression, and substance use disorder. We tested a sample of adults $\left(N=293\right)$ on the play-or-pass version of the IGT. We characterized behavior using a hierarchical Bayesian computational model, formalizing parameters underlying task behavior. With the model, we examined unique associations between IGT performance and lifetime diagnostic history of anxiety, depression, and substance use disorder. Anxiety, but not depression or substance use, was associated with higher punishment learning rates, posterior mean β=0.15, 95% CI [0.01, 0.28]. In addition, women showed lower punishment learning rates, posterior mean β=−0.17, 95% CI [− 0.3, − 0.03], and lower response bias, posterior mean β=−0.3, 95% CI [− 0.56, − 0.05]. The relation between punishment learning rates and history of anxiety was small; however, our findings were consistent with established findings in anxiety derived from self-report. In addition, only main effects of diagnostic status were examined; thus, future research should examine comorbidity between diagnoses on IGT performance. Overall, our findings are consistent with research showing that anxiety is associated with punishment avoidance. In addition, our behavioral findings with respect to gender are also consistent with previous research employing the IGT.

## Introduction

Reward and punishment learning are critical for adaptive functioning. Alterations in these processes are thought to contribute to multiple forms of psychopathology, including anxiety, depression, and substance use disorder (SUD; PVS Work Group, 2011; Sanislow et al., 2010). Studies examining reward and punishment learning in psychopathology typically focus on differences between individuals with and without specific diagnoses, leaving questions about whether impairments in these learning processes represent general deficits across disorders or unique deficits in a subset of disorders. Moreover, methodological challenges in the use of common behavioral measures for reward and punishment learning preclude refined understanding of which learning processes are impacted. Thus, this study examines the influence of common forms of psychopathology, anxiety, depression, and SUD on reward and punishment learning using computational modeling methods.

There are multiple assessments of reward and punishment learning; however, one of the more commonly used assessments is the Iowa Gambling Task (IGT; Bechara, 2007). The IGT is a complex decision-making task in which participants choose between four decks of cards across a series of trials (typically ~ 100 to 120 trials). Each card is associated with a hypothetical monetary gain (e.g., $100); however, some cards are associated with a hypothetical monetary loss presented alongside the gain, resulting in a net loss (e.g., + $100 & − $300 = − $200), gain (e.g., + $50 & − $25 = + $25), or nothing (e.g., + $50 & − $50 = $0) for that card. Decks vary in the frequency and magnitude of gains and losses such that two decks are advantageous, resulting in net monetary gains across the task, and the other two are disadvantageous, resulting in net monetary losses across the task. Participants are provided no information regarding the outcomes a priori; thus, they must learn from their choices within the task to identify which decks are advantageous and disadvantageous.

The IGT has long been used as a neuropsychological assessment of impaired reward and punishment learning. Early studies showed that damage to the ventromedial prefrontal cortex was associated with choosing disadvantageously on the task (Bechara et al., 1994). Later studies built on this work to show that other regions of the prefrontal cortex (e.g., the orbitofrontal cortex; Bolla et al., 2004) and outside of the prefrontal cortex (e.g., the amygdala; Gupta et al., 2011) are implicated in decision-making on the task (see also Li et al., 2010). Because many of the brain regions involved in the IGT overlap with regions highly implicated in mental illness, a number of studies have employed the IGT to study reward and punishment learning across multiple forms of psychopathology, including depression, anxiety, and substance use disorder.

Several studies have examined performance on the IGT among those with depression. Generally, depression is associated with poor IGT performance, indicating dysfunctional reward and punishment learning (Siqueira et al., 2018; Wang et al., 2024), although some mixed findings have been reported (McGovern et al., 2014; Smoski et al., 2008). Must et al. (2013) suggest that poor performance on the IGT among individuals with depression reflects a preference for immediate gains combined with an insensitivity to long-term losses (Must et al., 2013).

The relationship between anxiety and reward and punishment learning is less consistent; some studies show that anxiety is associated with suboptimal performance on the IGT (Li et al., 2024), and others show that anxiety is associated with better performance on the IGT (Mueller et al., 2010; Werner et al., 2009). Werner et al. (2009) suggest that these inconsistencies may reflect the influence of physiological arousal on IGT performance via an inverse U-shaped association (Yerkes & Dodson, 1908). Specifically, low anxiety may be associated with lower levels of physiological arousal, resulting in an insensitivity to outcomes in the task and thus poor performance. In contrast, high anxiety may be associated with higher levels of physiological arousal, interfering with learning from the outcomes in the task and resulting in poor performance. There is some support for Werner et al.’s hypothesis (de Visser et al., 2010; Zhang et al., 2015; although see Zhang et al., 2017), suggesting that the relation between anxiety and reward and punishment learning is more complex than simply categorizing performance in terms of better or worse decision-making.

Finally, substance use is generally associated with suboptimal performance on the IGT (Barry & Petry, 2008; Petry et al., 1998; Verdejo-Garcia et al., 2007), possibly related to impulsive decision-making (Bechara, 2005; Moffitt et al., 2011). Specifically, impulsive decision-making is frequently characterized as an insensitivity to the long-term consequences of one’s choices (Bickel et al., 2012), which may be reflected on the IGT as dysfunctional reward and punishment learning, resulting in poor performance on the task. As with depression and anxiety, however, there are some inconsistencies across studies examining substance abuse and IGT performance (Businelle et al., 2008). In sum, psychopathology generally appears to be related to poor performance on the IGT, despite some inconsistencies. The degree to which this poor performance reflects similar or unique learning processes across these forms of psychopathology, however, is still unclear.

A limitation of the IGT is that traditional scoring measures provide little insight regarding why groups may differ on the task (Ahn et al., 2016). Specifically, traditional scoring procedures typically involve summarizing choices for good and bad decks (e.g., choice proportions) across the whole task or across blocks of trials within the task (Case & Olino, 2020). Measures from traditional scoring procedures index sensitivity to the long-term outcomes associated with the good and bad decks (i.e., overall learning) but do not capture trial-level learning (Sullivan-Toole et al., 2022). This is problematic given the complexity of the task. For example, as noted above, the four decks in the IGT are typically categorized as good and bad, but decks within these categories vary in reward/punishment magnitude and frequency. These differences between decks within the good/bad categories result in different patterns of behavior, one of which being a sensitivity to win/loss frequency regardless of whether the deck is advantageous or disadvantageous in the long run (Chiu & Lin, 2007; Chiu et al., 2008; Steingroever et al., 2013a; Wetzels et al., 2010). Coupled with small sample sizes that are typical for clinical studies using the IGT (Mukherjee & Kable, 2014), aggregate measures like choice proportions may be inadequate to properly characterize how decision-making on the IGT relates to different forms of psychopathology.

Computational models based on reinforcement learning can provide a fine-grained analysis of IGT performance that could more adequately characterize reward and punishment learning in psychopathology (Ahn et al., 2016; Haines et al., 2018; Steingroever et al., 2013b). For example, Haines et al. (2018) developed the Outcome Representation Learning (ORL) model to decompose IGT performance into parameters representing reward and punishment learning rates, win frequency sensitivity, perseverative decision-making, and memory. Haines et al. showed that the ORL captures aspects of reward and punishment learning associated with substance abuse (e.g., lower punishment learning among heroin-users), beyond what could be derived from traditional scoring (see also Ahn et al., 2014). The ORL is particularly well-suited for the IGT because model parameters can account for key patterns of behavior among neurotypical samples (e.g., win/loss frequency sensitivity) and other patterns of behavior that may be unique to specific forms of psychopathology. For example, depression is often thought to be specifically associated with lower reward sensitivity in conjunction with heightened punishment sensitivity (Eshel & Roiser, 2010; Huys et al., 2013), whereas anxiety is thought to be specifically associated with heightened punishment sensitivity (Carver & White, 1994; Huang et al., 2017; Pike & Robinson, 2022). The ORL characterizes sensitivity to reward and punishment based on separate parameters (i.e., reward vs. punishment learning rates) and allows us to differentiate these sensitivities from win/loss frequency sensitivity which cannot be done with traditional scoring measures (i.e., good/bad play proportions). Thus, the ORL may be particularly useful for characterizing patterns of behavior on the IGT that are unique to different forms of psychopathology.

In the present study, we use an extension of the ORL model to characterize trait-like differences in reward and punishment learning across anxiety, depression, and substance use disorder. In a preliminary study with college students, Haynes et al. (2024) extended the ORL to characterize trial-level learning on the play-or-pass IGT, a version of the IGT that involves presenting cards one at a time and allows participants to “play” or “pass” on the card (Peters & Slovic, 2000). In addition, this version of the IGT involves combining the gains and losses from each card into a single outcome. These changes from the original IGT allow for distinguishing approach and avoidance behaviors because choices to play on each card are *not* mutually exclusive from playing on other cards and the outcomes are presented as either a gain, loss, or nothing, instead of a combination of outcomes (Cauffman et al., 2010). The play-or-pass ORL, built for the play-or-pass IGT, is a reinforcement learning model that captures trial-by-trial learning with four parameters: reward and punishment learning rates, win frequency sensitivity, and response bias. Haynes et al.’s preliminary study focused on model-development and thus has not been used to assess reward and punishment learning in the general population.

Our goal in the present study was to extend Haynes et al.’s (2024) work by examining reward and punishment learning in a sample of adults from the general population assessed on their lifetime history of psychopathology. Given the prior IGT literature illustrating differences in performance among individuals with and without anxiety, depression, and substance abuse, we examined differences between those with and without a history of these disorders using the play-or-pass ORL. The majority of participants in the sample with a history of psychopathology were in remission; thus, we examined differences in the ORL parameters as trait-like characteristics (Barry & Petry, 2008; Esfand et al., 2024). The model was fit to the IGT data using simultaneous predictors of anxiety, depression, and SUD to capture the unique association between the presence and absence of each of these disorders on reward and punishment learning. In addition, given differences in performance on the IGT across male and female participants (Zanini et al., 2024), we also include sex as a covariate in the model.

## Method and materials

Data came from a larger study focused on various facets of adolescent risk for psychopathology, examining parental history of psychopathology, adolescent reward sensitivity, and neural structure and function as factors influencing risk. We present baseline data from parents collected prior to the Covid-19 pandemic, and we focus on parents’ behavioral performance on the play-or-pass IGT and diagnostic data from the Structured Clinical Interview for the DSM-5 (SCID; First et al., 2016). All procedures were approved by the Temple University institutional review board (IRB Protocol #23,174 originally approved on 08/31/2015) and conducted in accordance with the Declaration of Helsinki. All participants provided informed consent, and their privacy has been upheld.

### Participants

Youth participants and their parents were recruited from the community within a 30-mile radius of a metropolitan university in the northeastern United States. English-speaking families with a child aged 9 − 10 or 12 − 13 years with at least one biological parent serving as their caregiver were eligible to participate. The present study focused on parents within these families $\left(N=296\right)$. Families were excluded from the larger study if their children were taking any psychotropic medications (except for ADHD medication), met diagnostic criteria for a serious neurological illness, or had learning or developmental disabilities at baseline. In addition, families with parental history of bipolar or psychotic spectrum disorder were excluded. Intellectual ability of adolescent children was examined at baseline using the Kaufman Brief Intelligence Test (KBIT-2; Naugle et al., 1993), and families with children whose estimates of overall intelligence levels fell two or more standard deviations below the mean were also excluded (KBIT-2 FSIQ < 70). Data for three parents were excluded: one parent responded on fewer than half of trials in the IGT; one did not finish the task; and one did not complete the SCID. Thus, the final sample size was 293 parents, inclusive of mothers and fathers, which hereafter will be referred to as female and male participants, respectively. Demographic characteristics are presented in Table 1.

### Procedure

Participants completed a range of behavioral, self-report, and neurological measures; however, we only describe the relevant measures for this study: the SCID, the play-or-pass IGT measured at baseline, and self-report measures assessing sensitivity to rewards and punishments. Participants were assessed for lifetime (i.e., past & current) history of internalizing and externalizing forms of psychopathology using the SCID. Across all parents, 93 (32%) had a history of anxiety (current n=65; past n=28), 92 (31%) had a history of depression (current n=10; past n=82), 85 (29%) had a history of substance use disorder (current n=9; past n=76), and 124 (43%) had no lifetime history of anxiety, depression, or substance use disorder. Table 2 presents full diagnostic characteristics, including comorbidities. Participants completed the play-or-pass IGT to assess reward and punishment learning (details below). Of the self-reports, we estimated correlations between IGT performance and scores from the Behavioral Inhibition/Behavioral Activation Scales (BIS/BAS; Carver & White, 1994). Some correlations were statistically significant, but all were small in magnitude (all rs<0.15). Thus, we present correlations between IGT performance and BIS/BAS scores in the supplement. Following the session, participants were compensated $95.

### Play‑or‑pass Iowa gambling task

The play-or-pass version of the IGT was administered using E-Prime Stimulus Presentation Software (Peters & Slovic, 2000; Schneider et al., 2002). All outcomes in the task were hypothetical. Participants began the task with a “bank” of $2,000 and were presented with four decks of cards. On each trial, a single deck was highlighted, and participants had the opportunity to “play” or “pass” on the highlighted deck. If a participant played on the deck, they would receive either a monetary gain, loss, or neither (i.e., $0 change) from the drawn card. If a participant passed on the deck, they moved onto the next trial. Decks varied in the frequency and magnitude of the gains and losses, resulting in long-run averaged expected values of − $25 for Decks A and B (i.e., the disadvantageous/bad decks) and $20 and $25 for Decks C and D, respectively (i.e., the advantageous/good decks). Further details of the distributions of these outcomes are displayed in Table 3. We used two versions of the task that differed only in the position of the decks, and participants were assigned to one of those versions using counterbalancing. Each deck was associated with a fixed sequence of cards and outcomes; however, participants were not informed of the payout distribution nor the sequence of decks and cards that would be presented. Thus, participants needed to sample cards from each deck to learn the outcomes associated with each deck. Trials ended if participants did not respond within 4 s which were scored as passing on the card. Finally, participants were told that their earnings in the task would be exchanged for a real cash bonus; however, all participants received $10 for completing the behavioral tasks.

### Data analysis

We used a hierarchical Bayesian approach to estimate parameters from the play-or-pass ORL model. The model was fit in Stan (v. 2.32; Stan Development Team, 2023b), a probabilistic programming language which estimates parameters using Hamiltonian Monte Carlo, a variant of Markov chain Monte Carlo (MCMC) to sample from high-dimensional probabilistic models. We used R (v. 4.2.2; R Core Team, 2022) to interface with Stan via the RStan package (v. 2.26.22; Stan Development Team, 2023a). We sampled the model using 4 chains, each with 5000 iterations with the first 1,000 iterations as warmup (Sullivan-Toole et al., 2022). After fitting the model, we checked for convergence of target distributions visually with trace-plots and for each parameter numerically with $\hat{R}$ values (Gelman & Rubin, 1992). $\hat{R}$ values were all below 1.1, indicating that the variance between chains did not outweigh the variance within chains (i.e., convergence). We performed posterior predictive checks by simulating data from the model and visually inspecting how well the model fit the data by comparing simulated data with the observed data. Finally, we performed parameter recovery diagnostics which showed adequate recovery (see supplement). The supplement, data, and code are available at https://osf.io/fg7sj/?view_only=cb5b3f32171c4309bf5438784c2d2e98.

#### Play‑or‑pass ORL model

We fit the play-or-pass Outcome-Representation Learning (PP-ORL) model (Haynes et al., 2024), a reinforcement learning model extended from Haines et al.’s (2018) ORL model developed for the original IGT. In the PP-ORL, choices to play or pass are modeled as a function of the value of playing on that deck using a Bernoulli likelihood function:

(1) $$Y_{j}\left(t\right)∼bernoulli\left(\frac{1}{1+exp\left(-V_{j}\left(t\right)\right)}\right)$$

where $Y_{j}\left(t\right)$ indicates whether a participant played $\left(Y_{j}\left(t\right)=1\right)$ versus passed $\left(Y_{j}\left(t\right)=0\right)$ when presented with deck j on trial t, and $V_{j}\left(t\right)$ is the value of playing when presented with deck j on trial t. This choice rule implies that the value of passing is always held constant at 0—only the value of playing is updated on a trial-by-trial basis. Specifically, after each choice, $V_{j}$ is updated according to the following equation:

(2) $$V_{j}\left(t+1\right)=EV_{j}\left(t+1\right)+EF_{j}\left(t+1\right)\cdot \beta f+\beta b$$

where $V_{j}\left(t+1\right)$ is the value of playing on deck j on the next trial (i.e., t+1), $EV_{j}\left(t+1\right)$ is the expected outcome value associated with playing or passing on deck j in the next trial, $EF_{j}\left(t+1\right)$ is the expected win frequency of playing or passing on deck j in the next trial, βf is a free parameter describing sensitivity to win frequency, and βb is a free parameter describing bias towards playing (when positive) or passing (when negative), regardless of the deck.

The expected outcome value $\left(EV_{j}\right)$ and expected win frequency $\left(EF_{j}\right)$ are updated from trial to trial based on the outcome received after playing on deck j. Specifically, the expected outcome value for the next trial is calculated by

(3) $$EV_{j}\left(t+1\right)=\left\{\begin{array}{cc} EV_{j}\left(t\right)+A_{rew}\cdot \left(x\left(t\right)-EV_{j}\left(t\right)\right) & if\;\;Y_{j}\left(t\right)=1\;\;and\;\;x\left(t\right)\geq 0\\ EV_{j}\left(t\right)+A_{pun}\cdot \left(x\left(t\right)-EV_{j}\left(t\right)\right) & if\;\;Y_{j}\left(t\right)=1\;\;and\;\;x\left(t\right)<0\\ EV_{j}\left(t\right) & if\;\;Y_{j}\left(t\right)=0 \end{array}\right.$$

where $EV_{j}\left(t\right)$ is the expected outcome value of playing on deck j in the current trial (i.e., t), $x\left(t\right)$ is the amount of the gain or loss, $A_{rew}$ is a free parameter describing learning rate for gains (i.e., when $x\left(t\right)>0$), and $A_{pun}$ is a free parameter describing learning rate for losses (i.e., when $x\left(t\right)<0$). The expected win frequency for the next trial is calculated by

(4) $$EF_{j}\left(t+1\right)=\left\{\begin{array}{cc} EF_{j}\left(t\right)+A_{rew}\cdot \left(sgn\left(x\left(t\right)\right)-EF_{j}\left(t\right)\right) & if\;\;Y_{j}\left(t\right)=1\;\;and\;\;x\left(t\right)\geq 0\\ EF_{j}\left(t\right)+A_{pun}\cdot \left(sgn\left(x\left(t\right)\right)-EF_{j}\left(t\right)\right) & if\;\;Y_{j}\left(t\right)=1\;\;and\;\;x\left(t\right)<0\\ EF_{j}\left(t\right) & if\;\;Y_{j}\left(t\right)=0 \end{array}\right.$$

where $EF_{j}\left(t\right)$ is the expected win frequency of playing on deck j in the current trial, $A_{rew}$ and $A_{pun}$ are as described above, and the $sgn\left(x\left(t\right)\right)$ term refers to the sign of the outcome (using the signum function).^1^ In addition to updating the expected win frequency of the current deck, the expected win frequencies for the other decks are also updated according to the following fictive updating rule:

(5) $$EF_{\prime j}\left(t+1\right)=\left\{\begin{array}{cc} EF_{\prime j}\left(t\right)+A_{pun}\cdot \left(\frac{-sgn\left(x\left(t\right)\right)}{C}-EF_{\prime j}\left(t\right)\right) & if\;\;Y_{j}\left(t\right)=1\;\;and\;\;x\left(t\right)\geq 0\\ EF_{\prime j}\left(t\right)+A_{rew}\cdot \left(\frac{-sgn\left(x\left(t\right)\right)}{C}-EF_{\prime j}\left(t\right)\right) & if\;\;Y_{j}\left(t\right)=1\;\;and\;\;x\left(t\right)<0\\ EF_{\prime j}\left(t\right) & if\;\;Y_{j}\left(t\right)=0 \end{array}\right.$$

where $EF_{’j}\left(t\right)$ is the expected win frequency of the other decks during the current trial, C is the number of other decks available (i.e., 3), and all other terms are as described above.

Combined, the PP-ORL model has four parameters to characterize patterns of behavior on the play-or-pass IGT: reward learning rates $\left(A_{\text{rew}}\right)$, punishment learning rates $\left(A_{\text{pun}}\right)$, win frequency sensitivity $\left(\text{β}f\right)$, and response bias $\left(\text{β}b\right)$. For illustration, we show model predictions for these parameters in the supplement (Fig. S1). In general, lower reward learning rates $\left(A_{\text{rew}}\right)$ are associated with playing less frequently on the bad decks (i.e., deck A & B), because participants do not approach the higher-magnitude gains on those decks (Table 3). Higher punishment learning rates $\left(A_{\text{pun}}\right)$ are also associated with playing less frequently on the bad decks, but, in contrast, because participants avoid the losses on those decks. This is particularly the case for deck B, which contains the largest single-trial loss (− $,1150), and the model predicts that avoidance is greatest following the first encounter with that loss. Higher win frequency sensitivities $\left(\text{β}f\right)$ are associated with playing more frequently on decks with more wins (decks B & D) and less frequently on decks with fewer wins and more losses (i.e., decks A & C). In particular, higher win frequency sensitivities are associated with playing less on deck A, because this deck contains relatively few wins and the most losses compared with the other decks. Finally, higher response bias $\left(\text{β}b\right)$ is associated with playing more frequently across all decks. Thus, parameters from the PP-ORL allow us to capture individual-differences in task performance.

#### Parameterization for group‑comparisons

To make comparisons across diagnostic groups, parameters were estimated using an approach similar to a multiple regression. Specifically, each parameter was estimated with the following equation:

(6) $$\gamma_{i}={µ}_{i}+z_{i}\cdot \sigma$$

where $\gamma_{i}$ is the ORL parameter (i.e., $\text{`}A_{rew}$, $`A_{pun}$, βf, βb) for participant i; $\mu_{i}$ represents a group-level model (discussed below); $z_{i}$ is the standardized (i.e., z-scored) subject-specific effect of participant i; and σ is the overall standard deviation for the ORL parameter, which captures the hierarchical pooling of person-level estimates to the group-level mean. The parameters, $`A_{rew}$ and $`A_{pun}$, were transformed using the cumulative distribution function for the normal distribution (with scale=1), allowing $A_{rew}$ and $A_{pun}$ to be bounded between 0 and 1 (Haines et al., 2018). The group-level model, $\mu_{i}$, was estimated as the following:

(7) $${µ}_{i}=\beta_{0}+\beta_{Anx}X_{Anx,i}+\beta_{Dep}X_{Dep,i}+\beta_{SUD}X_{SUD,i}+\beta_{Sex}X_{Sex,i}$$

where $\beta_{0}$ is the average value of the parameter for male participants (reference sex) without anxiety, depression, or substance use disorder (i.e., when all other parameters are 0; cf. intercept); $\beta_{Anx}$, $\beta_{Dep}$, and $\beta_{SUD}$ are the effects of a history of anxiety, depression, and substance use disorder, respectively, on the parameter, $\beta_{Sex}$ characterizes sex-differences on the parameter; and $X_{Anx,i}$, $X_{Dep,i}$, and $X_{SUD,i}$ are indicator variables for each diagnosis: presence coded as X=1 and absence coded as X=0. Finally, $X_{Sex,i}$ is the indicator for male $\left(X=0\right)$ and female $\left(X=1\right)$ participants. This parameterization allowed us to capture the unique effect for the presence versus absence of each diagnosis on the parameter of interest while also controlling for overall differences in performance as a function of sex. For example, $\beta_{Anx}$ for $`A_{pun}$ captures the effect of a lifetime history of anxiety on punishment learning rates above and beyond (i.e., controlling for) the effects of a lifetime history of depression $\left(\beta_{Dep}\right)$ and substance use disorder $\left(\beta_{SUD}\right)$, and for being a male or female participant. Sex-differences were also examined as a post-hoc analysis.

#### Hierarchical Bayesian estimation

To estimate parameters, we used a hierarchical Bayesian approach with weakly informative priors. For coefficients in the group-level model (i.e., $\mu_{i}$), we used the following priors:

$$\beta_{0},\beta_{Anx},\beta_{Dep},\beta_{SUD},\beta_{Sex}∼Normal\left(0,1\right).$$

The priors on the group-level standard deviations were

$$\sigma ∼Half-Normal\left(0,2\right)$$

for $`A_{\text{rew}}$ and $`A_{pun}$, and

$$\sigma ∼Half-Cauchy\left(1\right)$$

for βf and βb. Finally, we used the following priors for the person-level parameters:

$$z_{i}∼Normal\left(0,1\right).$$

This is the noncentered parametrization of the person-level parameters, used to increase efficiency, but is functionally equivalent to the more commonly presented centered parameterization for person-level parameters in hierarchical models: $\gamma_{i}\sim \text{Normal}\left(\mu ,\sigma \right)=\mu +z_{i}\;\cdot \;\sigma$ (i.e., Eq. 6), where $z_{i}\sim \text{Normal}\left(0,\;1\right)$.

Because we used a Bayesian approach, we based inferences of parameters on evaluating the posterior distributions. For visually inspecting trends in the data, we present observed and model-predicted group- and person-level play proportions as well as summaries of the group-level parameters. We focus on the simultaneous main effects of anxiety, depression, substance use disorder (SUD), and sex ($\beta_{Anx}$, $\beta_{Dep}$, $\beta_{SUD}$, $\beta_{Sex}$, respectively) for each ORL parameter. Posterior distributions of βs in which the 95% credible interval (CI) did *not* overlap with 0 are considered statistically significant.

## Results

**Fig. 1:**
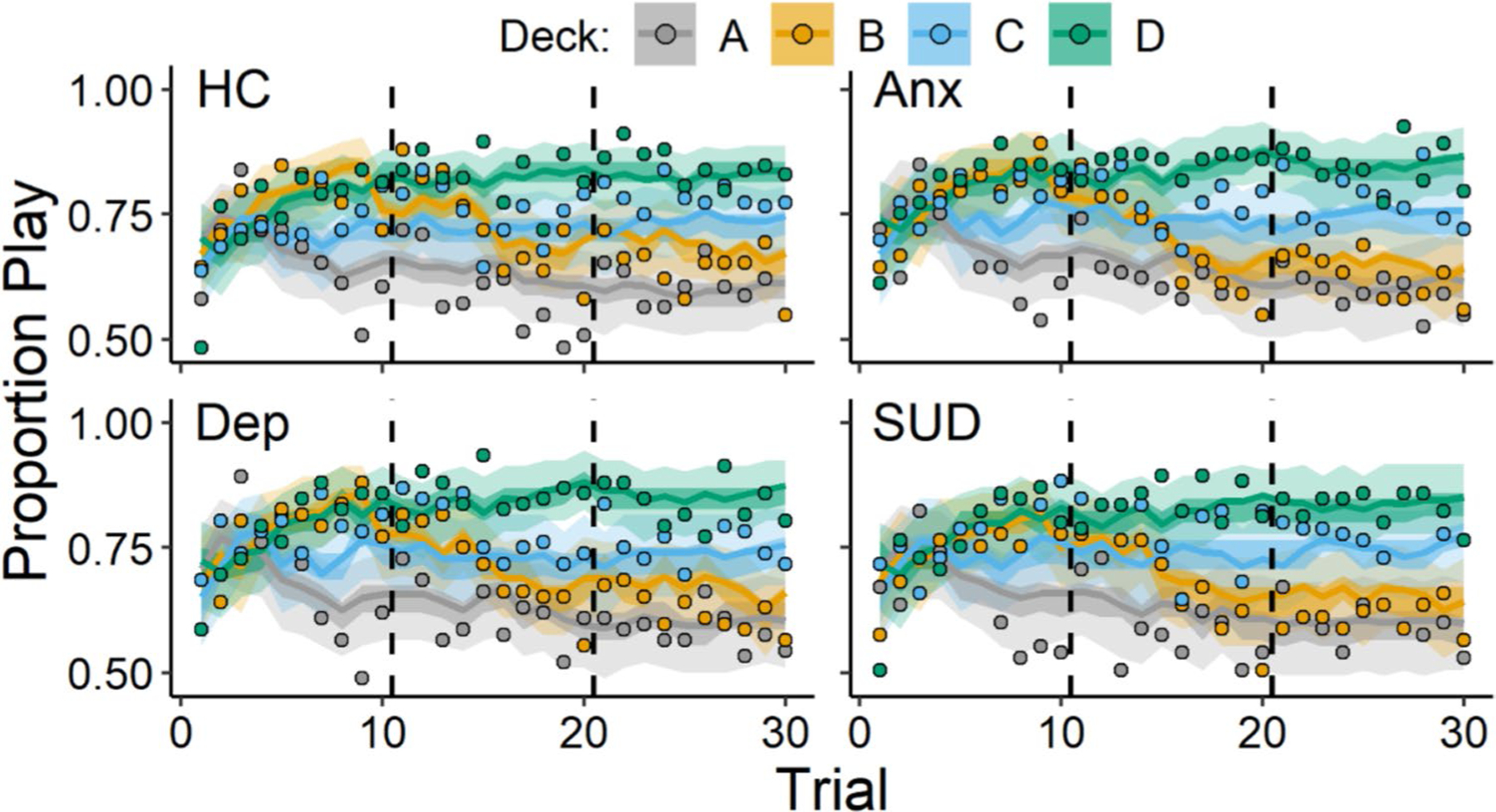
Play proportions for diagnostic group comparisons. *Note.* Group-level mean play proportions across trials for each deck among individuals with a lifetime history of anxiety (Anx), depression (Dep), substance use disorder (SUD), and those without these diagnoses (i.e., healthy controls; HC). Datapoints represent observed play proportions and lines represent ORL model-predicted play proportions. Error bands represent 50% (darker) and 95% (lighter) credible intervals from the ORL

Overall, participants showed learning of the advantageous and disadvantageous decks such that at the beginning of the task, most participants played on each deck equally often, but, by the end of the task, participants played on the good decks more frequently than on the bad decks. Choice data, illustrating these patterns of behavior, are displayed in Figs. 1 and 3, grouped according to diagnostic history (Fig. 1) and sex (Fig. 3). Parameters from the ORL model characterized these patterns of behavior, and the posterior distributions of these parameters are summarized in Table 4. Next, we describe group-differences with respect to psychopathology and sex, captured by these posterior distributions.

**Fig. 2:**
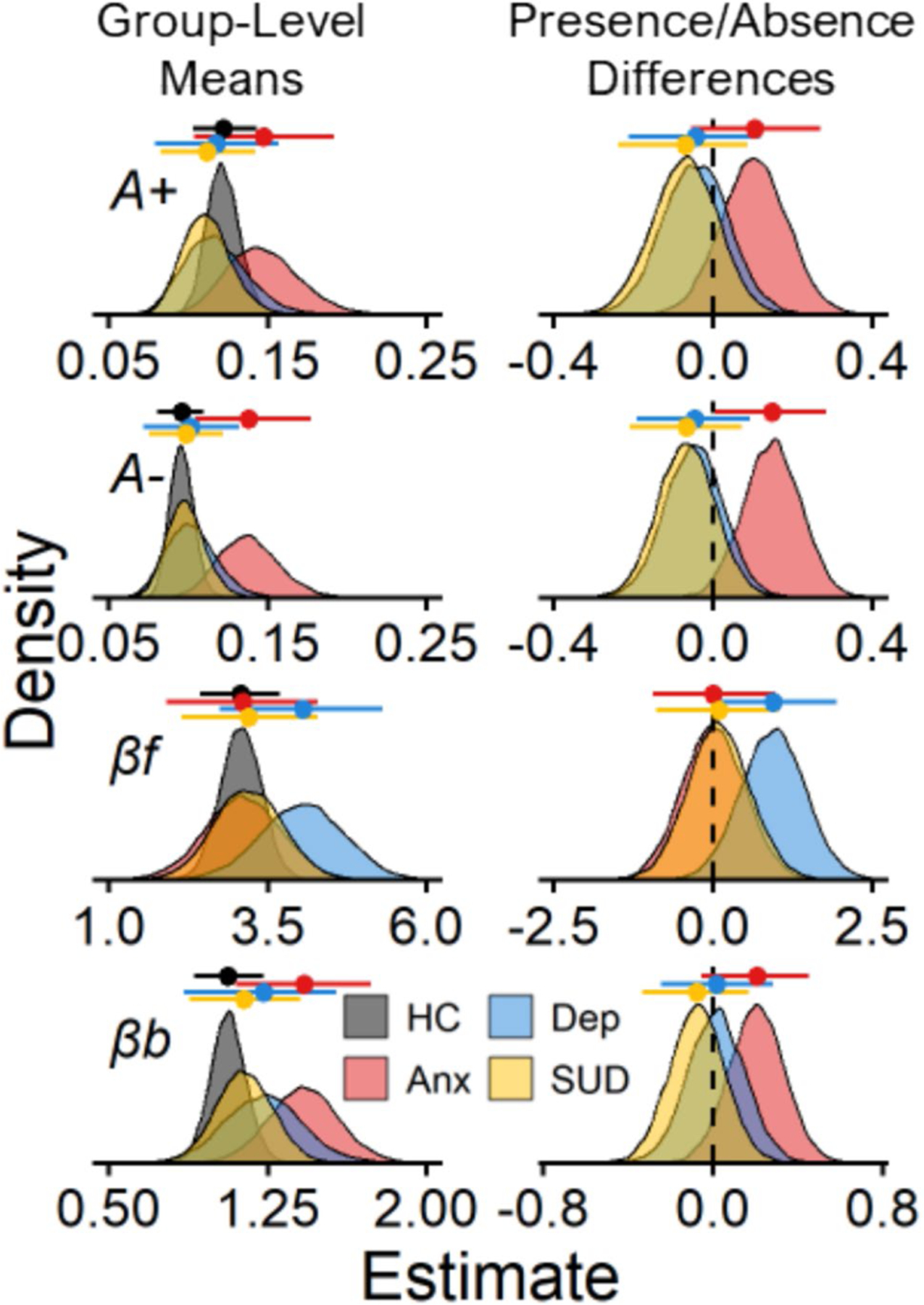
Posterior distributions for diagnostic group-comparisons. *Note.* Posterior distributions of reward learning rates $\left(A+\right)$, punishment learning rates $\left(A-\right)$, win frequency sensitivities $\left(\beta f\right)$, and bias parameters $\left(\beta b\right)$ from the PP-ORL model. The left column shows group-level means for each parameter (rows) across those with a history of anxiety (Anx), depression (Dep), substance use disorder (SUD), and those without these disorders (i.e., healthy controls; HC). The right column shows mean-differences between the presence and absence of a history of anxiety, depression, and substance use disorder for each parameter, where a higher mean-difference corresponds to elevated parameter values among the diagnostic history group. Data points with lines above each distribution represent posterior means ± 95% credible intervals

Figures 1 and 2 display results for the comparisons between the presence and absence of a history of anxiety, depression, and substance use disorder. In Fig. 1, we show the observed and ORL model-predicted trial-level play proportions for each diagnostic history group. In Fig. 2, we show the posterior distributions of the group-level means for each ORL parameter with respect to diagnostic history. Overall, participants with a history of anxiety showed higher punishment learning rates $\left(A\text{-}\right)$ than participants without a history of anxiety (posterior mean $\beta_{Anx}=0.15$, 95% CI [0.01, 0.28]). The higher punishment learning rates among individuals with a history of anxiety characterize how these participants responded differently towards losses on the task. For example, participants with a history of anxiety tended to show a more rapid decline in playing on deck B than those without a history of anxiety, beginning in the second block of trials (trials 10–20) after they would encounter the large loss on that deck (− $1150; Fig. 1). A more detailed comparison of these patterns of behavior between those with and without a history of anxiety on deck B is displayed in the supplement (Figs. S2 and S3).

Elevated punishment learning rates were the only significant effect for the diagnostic predictors; however, as Fig. 2 shows, other ORL parameters tended to be related to diagnostic history. Specifically, anxiety was associated with higher response bias and somewhat higher reward learning rates, with 94% and 89% of the $\beta_{Anx}$ posterior distributions located above 0 for βb and $A_{rew}$, respectively. The response bias parameter $\left(\beta b\right)$ captures a higher likelihood of playing, regardless of the deck, which may have been reflected by those with anxiety playing more during the first block of trials (i.e., trials 1–10; M play proportion = 0.76) than those without anxiety (M play proportion = 0.73). In addition, depression was associated with higher win frequency sensitivities, with 97% of the $\beta_{Dep}$ posterior distribution located above 0 for βf. For those with depression, the higher win frequency sensitivity may have reflected a bias away from deck A which consisted of the highest number of losses.^2^ Between the first and last block of trials, those with depression showed steeper decreases in choosing deck A (Block 3 – Block 1 $M_{\text{Diff}}=-0.09$) compared with those without depression (Block 3 – Block 1 $M_{\text{Diff}}=-0.06$). Thus, the ORL may capture additional aspects of decision-making related to anxiety as well as with depression.

**Fig. 3:**
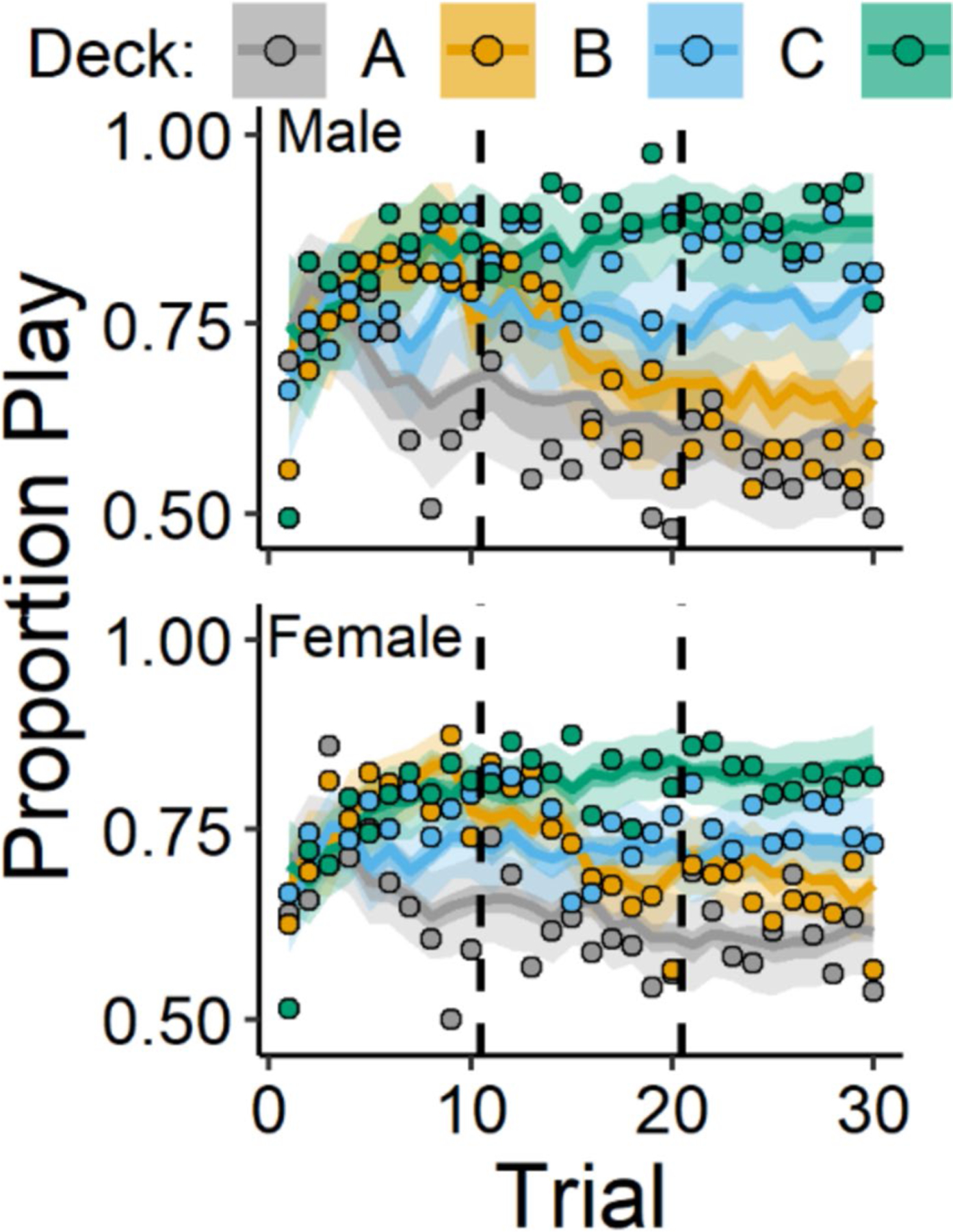
Play proportions for male and female participants. *Note.* Group-level mean play proportions across trials for each deck among male (top) and female (bottom) participants. Datapoints represent observed play proportions and lines represent ORL model-predicted play proportions. Error bands represent 50% (darker) and 95% (lighter) credible intervals from the ORL

**Fig. 4:**
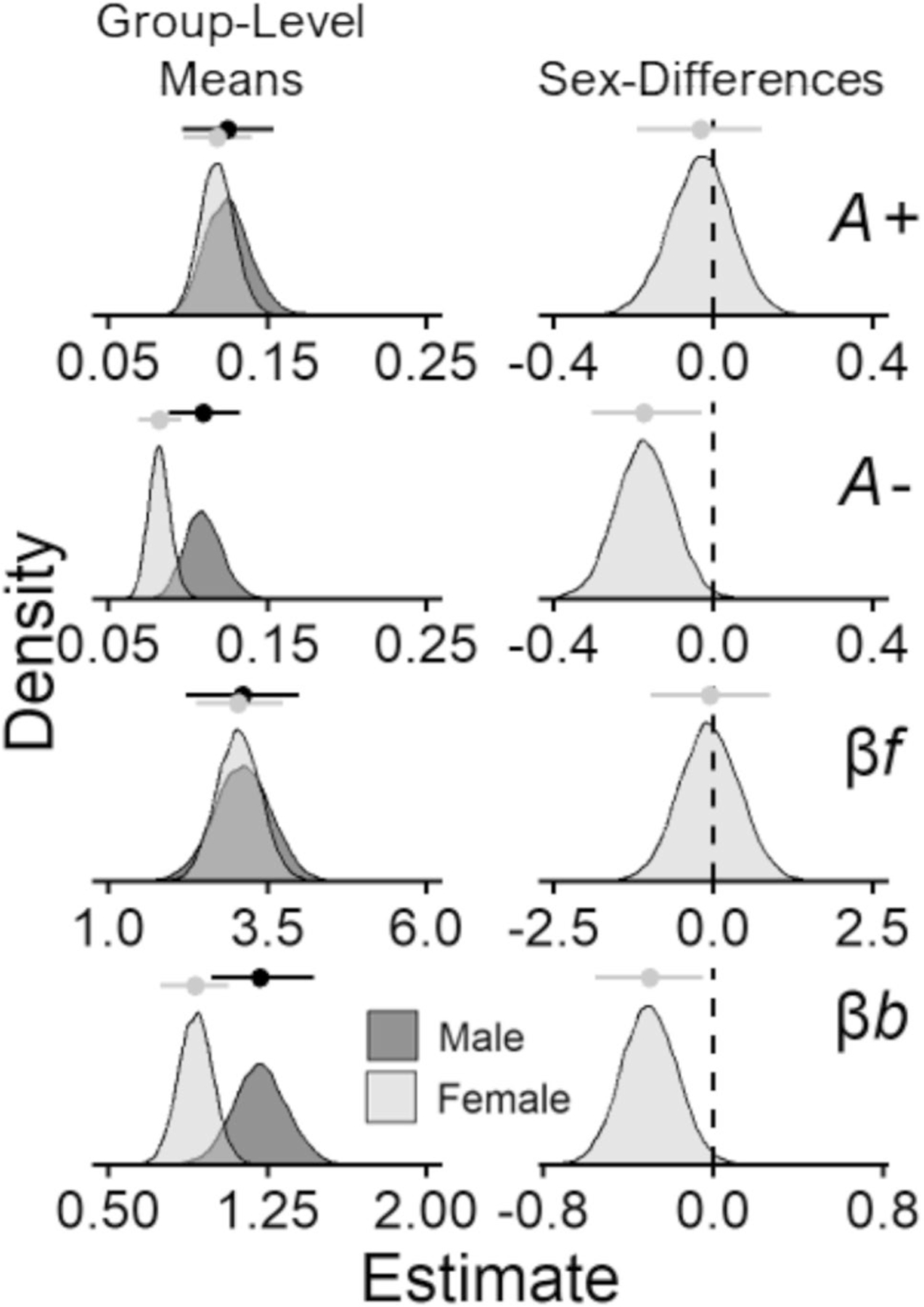
Posterior distributions for comparisons between male & female participants. *Note.* Posterior distributions of reward learning rates $\left(A+\right)$, punishment learning rates $\left(A-\right)$, win frequency sensitivities $\left(\beta f\right)$, and bias parameters $\left(\beta b\right)$ from the PP-ORL model. The left column shows group-level means for each parameter (rows) across male/female participants. The right column shows mean-differences between male and female participants, where a higher mean-difference corresponds to elevated parameter values among female participants. Data points with lines above each distribution represent posterior means ± 95% credible intervals

Figure 3 (play proportions) and Fig. 4 (posterior distributions) display results for the comparisons between male and female participants. Overall, female participants showed lower punishment learning rates (posterior mean $\beta_{Sex}=-0.17$, 95% CI [− 0.3, − 0.03]) and lower response bias (posterior mean $\beta_{Sex}=\;-0.3$, 95% CI [− 0.56, − 0.05]) than male participants. These results capture a pattern of behavior in which female participants played less frequently on the good decks and more frequently on the bad decks than male participants. This is illustrated in Fig. 3 wherein female participants show less separation in playing on the good decks (C & D) versus bad decks (A & B) than male participants. These sex differences are also displayed in more detail in the supplemental file (Fig. S5).

## Discussion

We used the play-or-pass version of the Iowa Gambling Task (IGT) to characterize reward and punishment learning across three common forms of psychopathology: anxiety, depression, and substance use disorder. Learning was characterized using the play-or-pass Outcome Representation Learning (ORL) model, a reinforcement learning model previously developed specifically for this version of the IGT (Haynes et al., 2024). For the diagnostic group-comparisons, our main finding was that individuals with a history of anxiety showed higher punishment learning rates. For the comparisons between sexes, we found that female participants had lower punishment learning rates and lower response bias than male participants. Differences in these ORL parameters reflect differences in specific patterns of behavior within the IGT that were uniquely associated with the presence versus absence of a history of anxiety and with male versus female participants. We discuss each finding in turn below.

Our main finding from the diagnostic comparisons was higher punishment learning rates in anxiety. This is consistent with a meta-analysis conducted by Pike and Robinson (2022), which showed elevated punishment learning rates in anxiety and other mood disorders. Brown et al. (2023) suggest that elevated punishment learning rates in anxiety specifically reflects a heightened aversion to uncertain losses (see also Bishop & Gagne, 2018). Given that performance on the IGT is a form of probabilistic learning from uncertain outcomes (Brevers et al., 2013), the higher punishment learning rates observed in the present study may be capturing this feature of anxiety. Unlike Pike & Robinson’s meta-analysis, we did not find that anxiety was associated with *reduced* reward learning rates. Instead, we found slightly elevated reward learning rates in anxiety. Pike & Robinson show that the relation between anxiety and reward learning rates is quite small (Cohen’s d = 0.02; Cohen, 1994; Funder & Ozer, 2019); thus, the play-or-pass ORL may not be sensitive enough to capture this small difference between those with and without anxiety. Importantly, our finding that increased punishment learning rates were associated with anxiety is consistent with the larger body of literature suggesting that anxiety is characterized by a heightened sensitivity to punishing outcomes (Nettle & Bateson, 2012).^3^ Neurobiologically, this heightened sensitivity to punishment in anxiety may be tied to limbic activation (e.g., insula & habenula; Huggins et al., 2021) associated with negative prediction errors (Garrison et al., 2013).

In addition to the elevated punishment learning rates in anxiety, other ORL parameters showed small associations with diagnostic history. Anxiety was associated with higher response bias and somewhat higher reward learning rates. The higher response bias in particular may have captured a small trend in which those with anxiety played more during the first block of trials than those without anxiety. This could reflect higher information-seeking associated with anxiety (e.g., as in obsessive compulsive disorder; Morein-Zamir et al., 2018), such that playing more at the beginning of the task provides more information regarding which decks are good and bad. In addition, win frequency sensitivity tended to be higher among those with depression which may have reflected a bias away from the deck with the most frequent losses (i.e., deck A). Heightened sensitivity to loss is considered a characteristic of depression (Eshel & Roiser, 2010); thus, our data could suggest that a heightened sensitivity to the frequency of losses is particularly relevant for depression. Given that this finding, and the others involving anxiety (i.e., reward learning rates & response bias), were not significant according to our statistical criteria (i.e., 95% HDIs above/below 0 for the β coefficients), and that the behavioral differences were rather small, we urge caution in interpreting these findings.

Besides the differences between diagnostic groups, we also found that female participants had lower punishment learning rates and lower response bias than male participants. This reflects a pattern of behavior in which female participants played on the good decks less frequently, and on the bad decks more frequently, than male participants. Although the magnitude of sex-related differences in performance on the IGT may be modest (Grissom & Reyes, 2019), our findings are consistent with a recent meta-analysis in which, on average, men choose good decks over bad decks more frequently than women on the original IGT (Zanini et al., 2024).^4^ Research with rats using the rodent-IGT supports this finding (van den Bos et al., 2012), although not as consistently when other gambling tasks are considered (Orsini & Setlow, 2017).

The mechanisms underlying these sex-differences are still unclear. Reviews by van den Bos et al. (2013) and Grissom and Reyes (2019) suggest that, behaviorally, women/female participants prioritize avoiding more frequent losses over avoiding losses of large magnitude, compared with men/male participants, which may underlie these sex-differences. This could explain why female participants tend to choose the bad deck with the largest but most infrequent losses more often than men on the IGT (Fig. 3; Overman & Pierce, 2013). However, because the IGT confounds magnitude with frequency of both gains and losses, it is difficult to tease these processes apart to clarify these sex-differences. Neurobiologically, sex-differences on the IGT may be due to functional hemispheric asymmetry between the sexes. For example, Bolla et al. (2004) found that men, compared with women, showed better IGT performance and greater right-lateralized activation in the OFC and DLPFC, regions that have been linked to monetary punishment (O’Doherty et al., 2001) and avoidance behavior (Berkman & Lieberman, 2010), respectively. Thus, greater right-lateralized prefrontal activation in these regions may facilitate punishment avoidance, which is necessary for optimal performance on the IGT. An important next step for using the play-or-pass ORL to characterize these sex-differences will be to examine whether punishment learning rates and response bias are associated with differential neural activation between the sexes. Importantly, however, our findings suggest that the play-or-pass ORL can capture sex differences on the IGT that characterize patterns of behavior consistent with the broader literature on sex-differences in the IGT (Zanini et al., 2024).

This study has multiple strengths. Our sample size allowed us to detect differences in reward and punishment learning among the diagnostic groups that may have otherwise been too small to detect. This is important given that there are several inconsistencies in the IGT literature (Buelow & Barnhart, 2018; McGovern et al., 2014; Mueller et al., 2010). Thus, adequately powered studies, with larger sample sizes, are necessary to be confident in the results from those studies. Second, we used a Bayesian computational modeling approach which provided a more fine-grained analysis of learning on the play-or-pass IGT and allowed us to characterize choice on the task as a function of multiple behavioral processes. Finally, because we included multiple forms of psychopathology as simultaneous predictors, we were able to identify unique associations for individual forms of psychopathology. Relatedly, we controlled for sex-differences on the task, and our behavioral analysis of task performance between male and female participants showed consistent patterns with prior research (Zanini et al., 2024).

Despite these strengths, our study also has limitations. First, our categorization of participant psychopathology was based on lifetime histories, and with the exception of anxiety, there were few individuals with current diagnoses. We conducted a follow-up analysis to examine differences between those with current $\left(n=65\right)$ and remitted $\left(n=28\right)$ anxiety but found no differences in the ORL parameters between these groups (see supplement for further details), indicating the elevated punishment learning rates is a trait-like characteristic of anxiety. This follow-up analysis is likely underpowered; thus, we urge caution in interpreting those findings. In addition, given the small sample sizes of those with current depression $\left(n=10\right)$ and SUD $\left(n=9\right)$, we did not conduct follow-up analyses owing to lack of statistical power. Therefore, future research would benefit from having larger samples of individuals with both past and current diagnoses to test whether differences in reward and punishment learning between those with and without these disorders reflect state or trait effects.

Second, because the sample was composed of parent dyads, some of whom both completed the IGT, it is possible that nonindependence could have affected our results, particularly with respect to the differences in punishment learning rates with anxiety. People with affective disorders, including anxiety, are more likely to mate with others who share similar affective phenotypes (a form of assortative mating; Mathews, 2001; Nordsletten et al., 2016), and this lack of independence can bias statistical analyses (Kenny, 1995). To probe this issue, we examined the correlation between mother and father $\left(n=67\;\;\text{dyads}\right)$ punishment learning rates. This correlation was positive, but weak $\left(r=0.12\right)$, indicating dependence may not be particularly problematic in our data. In addition, we conducted a sensitivity analysis in which we randomly removed one participant from each dyad and reran our analyses. This analysis replicated the finding that anxiety is associated with higher punishment learning rates (posterior mean $\beta_{Anx}=0.17$, 95% CI [0.03, 0.31]; see supplement for further details). This is particularly important, because although the $\beta_{Anx}$ estimate for punishment learning rates was significant in the main analysis, the lower bound of the 95% credible interval was only marginally above 0, suggesting that differences in punishment learning rates between those with and without anxiety may be marginal. It is also worth noting, however, that our pattern of findings is consistent when examining self-report measures such that punishment learning rates were positively associated with BIS scores (see supplement), which are elevated among individuals with anxiety (Carver & White, 1994). Thus, although further research is necessary to replicate our findings, our follow-up analyses (i.e., the sensitivity analysis & correlations with self-reports) suggest that our observation of elevated punishment learning rates in anxiety is reliable.

A final limitation to consider is that we only assessed the main effects of sex and each form of psychopathology on IGT performance but not how interactions between different disorders (i.e., comorbidities) and between the disorders and sex affected performance. Although we had a well-sized sample for main effects, power to detect interactions requires large samples (Baranger et al., 2023), particularly for crossed effects like comorbidity. Furthermore, given the differential prevalence rates of psychopathology across the sexes (Hartung & Lefler, 2019), more balanced sample sizes of male and female participants with each disorder would allow for examination of how psychopathology specifically influences behavioral processes that vary according to sex (e.g., punishment learning). Such work will be important when developing treatments tailored according to individual differences in psychopathology.

In sum, our findings validate the play-or-pass ORL model for capturing clinically relevant aspects of reward and punishment learning. We found that punishment learning rates were elevated among individuals with a history of anxiety, supporting prior research employing reinforcement learning models to understand decision-making in anxiety. We did not, however, find that reward and punishment learning were unique to a history of depression or substance use disorder, unlike in prior research (Haines et al., 2018; Siqueira et al., 2018). Finally, the ORL model identified sex-differences in punishment learning that characterized patterns of decision-making between male and female participants that are consistent with prior research.

## Supplementary Material

Complete supplement

The online version contains supplementary material available at https://doi.org/10.3758/s13415-025-01331-y.

## Figures and Tables

**Table 1:** Demographics characteristics

	N=293
*M* (*SD*) age (years)	39.88 (6.99)
Male	77 (26%)
Female	216 (74%)
Race	
Asian	11 (4%)
Black or African American	4 (1%)
Pacific Islander	15 (5%)
White	107 (37%)
Not disclosed	156 (53%)
Ethnicity	
Hispanic	21 (7%)
Non-Hispanic	265 (90%)
Not disclosed	7 (2%)

**Table 2:** Diagnostic characteristics

Diagnosis	Anxiety	Depression	SUD
Anxiety	33	—	—
Depression	28	23	—
SUD	6	15	38
All three	26	26	26
Total	93	92	85

Singular diagnoses are given by intersections of the same diagnosis and comorbid diagnoses are given by intersections of different diagnoses

**Table 3:** Task-wide descriptive statistics of decks

	Deck			
Statistic	A	B	C	D
Mean	− 25	− 25	20	25
Sum	− 750	− 750	600	750
Gain frequency	15	27	15	27
$0 Frequency	0	0	9	0
Loss frequency	15	3	6	3
Highest gain	100	100	50	50
Highest loss	− 250	− 1,150	− 25	− 200

**Table 4:** Posterior means and 95% credible intervals for model parameters

ORL model parameter	Group-level model parameter				
	$\beta_{0}$	$\beta_{\text{Anx}}$	$\beta_{\text{Dep}}$	$\beta_{\text{SUD}}$	$\beta_{\text{Sex}}$
A+	− 1.2 [− 1.34, − 1.05]	0.10 [− 0.06, 0.27]	− 0.04 [− 0.21, 0.12]	− 0.07 [− 0.24, 0.09]	− 0.03 [− 0.19, 0.12]
A−	− 1.32 [− 1.46, − 1.20]	**0.15 [0.01, 0.28]**	− 0.05 [− 0.19, 0.09]	− 0.07 [− 0.21, 0.07]	**− 0.17 [− 0.3, − 0.03]**
βf	3.11 [2.22, 3.99]	0.00 [− 0.95, 0.98]	0.94 [− 0.05, 1.93]	0.09 [− 0.89, 1.03]	− 0.06 [− 0.99, 0.89]
βb	1.22 [0.99, 1.47]	0.2 [− 0.06, 0.45]	0.01 [− 0.25, 0.28]	− 0.08 [− 0.33, 0.17]	**− 0.3 [− 0.56, − 0.05]**

Bolding represents the β-comparisons in which the 95% CI did *not* overlap with 0. For $\beta_{\text{Sex}}$, male participants were the reference category

## Data Availability

Study materials are available upon request and data are available at https://osf.io/fg7sj/?view_only=cb5b3f32171c4309bf5438784c2d2e98.

## Footnotes

1 For $x\left(t\right)>0$, $sgn\left(x\left(t\right)\right)=+1$; for $x\left(t\right)=0$, $sgn\left(x\left(t\right)\right)=0$; for $x\left(t\right)<0$, $sgn\left(x\left(t\right)\right)=-1$.

2 There were no systematic differences in playing between those with and without depression for the decks with more frequent wins (decks B & D).

3 We use the term “sensitivity” to refer to the general effect of an outcome on behavior. It should be noted that some researchers use “sensitivity” to describe valuation of outcomes and specifically distinguish it from learning rates that represent how much learning occurs following outcomes (Pike & Robinson, 2022).

4 We use the terms employed by authors related to sex and gender. Although sex and gender are intertwined (Hyde et al., 2019), we urge caution is interpreting findings across studies focusing on sex vs. gender.

## Code availability

Code is available at https://osf.io/fg7sj/?view_only=cb5b3f32171c4309bf5438784c2d2e98.

## References

[R1] Ahn WY, Dai J, Vassileva J, Busemeyer JR, & Stout JC (2016). Computational modeling for addiction medicine: From cognitive models to clinical applications. Progress in Brain Research, 224, 53–65. 10.1016/bs.pbr.2015.07.032 26822353

[R2] Ahn WY, Vasilev G, Lee SH, Busemeyer JR, Kruschke JK, Bechara A, & Vassileva J (2014). Decision-making in stimulant and opiate addicts in protracted abstinence: Evidence from computational modeling with pure users. Frontiers in Psychology, 5, 849. 10.3389/fpsyg.2014.00849 25161631 PMC4129374

[R3] Baranger DAA, Finsaas MC, Goldstein BL, Vize CE, Lynam DR, & Olino TM (2023). Tutorial: Power analyses for interaction effects in cross-sectional regressions. Advances in Methods and Practices in Psychological Science, 6(3), 25152459231187532. 10.1177/25152459231187531 PMC12341451 40799847

[R4] Barry D, & Petry NM (2008). Predictors of decision-making on the Iowa Gambling Task: Independent effects of lifetime history of substance use disorders and performance on the Trail Making Test. Brain and Cognition, 66(3), 243–252. 10.1016/j.bandc.2007.09.001 17942206 PMC2292486

[R5] Bechara A (2005). Decision making, impulse control and loss of willpower to resist drugs: A neurocognitive perspective. Nature Neuroscience, 8(11), 1458–1463. 10.1038/nn1584 16251988

[R6] Bechara A (2007). Iowa gambling task professional manual. In Psychological Assessment Resources. Lutz.

[R7] Bechara A, Damasio AR, Damasio H, & Anderson SW (1994). Insensitivity to future consequences following damage to human prefrontal cortex. Cognition, 50(1–3), 7–15. 10.1016/0010-0277(94)90018-3 8039375

[R8] Berkman ET, & Lieberman MD (2010). Approaching the bad and avoiding the good: Lateral prefrontal cortical asymmetry distinguishes between action and valence. Journal of Cognitive Neuroscience, 22(9), 1970–1979. 10.1162/jocn.2009.21317 19642879 PMC3025747

[R9] Bickel WK, Jarmolowicz DP, Mueller ET, Koffarnus MN, & Gatchalian KM (2012). Excessive discounting of delayed reinforcers as a trans-disease process contributing to addiction and other disease-related vulnerabilities: Emerging evidence. Pharmacology & Therapeutics, 134(3), 287–297. 10.1016/j.pharmthera.2012.02.004 22387232 PMC3329584

[R10] Bishop SJ, & Gagne C (2018). Anxiety, depression, and decision making: A computational perspective. Annual Review of Neuroscience, 41, 371–388. 10.1146/annurev-neuro-080317-062007 29709209

[R11] Bolla KI, Eldreth DA, Matochik JA, & Cadet JL (2004). Sex-related differences in a gambling task and its neurological correlates. Cerebral Cortex, 14(11), 1226–1232. https://doi-org.libproxy.temple.edu/10.1093/cercor/bhh083 15142963 10.1093/cercor/bhh083

[R12] Brevers D, Bechara A, Cleeremans A, & Noël X (2013). Iowa Gambling Task (IGT): Twenty years after–gambling disorder and IGT. Frontiers in Psychology, 4, 665. 10.3389/fpsyg.2013.00665 24137138 PMC3786255

[R13] Brown VM, Price R, & Dombrovski AY (2023). Anxiety as a disorder of uncertainty: Implications for understanding maladaptive anxiety, anxious avoidance, and exposure therapy. Cognitive, Affective, & Behavioral Neuroscience, 23(3), 844–868. 10.3758/s13415-023-01080-w PMC10475148 36869259

[R14] Buelow MT, & Barnhart WR (2018). Test-retest reliability of common behavioral decision making tasks. Archives of Clinical Neuropsychology, 33(1), 125–129. 10.1093/arclin/acx038 28430836

[R15] Businelle MS, Apperson MR, Kendzor DE, Terlecki MA, & Copeland AL (2008). The relative impact of nicotine dependence, other substance dependence, and gender on Bechara Gambling Task performance. Experimental and Clinical Psychopharmacology, 16(6), 513. 10.1037/a0013510 19086772

[R16] Carver CS, & White TL (1994). Behavioral inhibition, behavioral activation, and affective responses to impending reward and punishment: The BIS/BAS scales. Journal of Personality and Social Psychology, 67(2), 319. 10.1037/0022-3514.67.2.319

[R17] Case JA, & Olino TM (2020). Approach and avoidance patterns in reward learning across domains: An initial examination of the Social Iowa Gambling Task. Behaviour Research and Therapy, 125, 103547. 10.1016/j.brat.2019.103547 31954996

[R18] Cauffman E, Shulman EP, Steinberg L, Claus E, Banich MT, Graham S, & Woolard J (2010). Age differences in affective decision making as indexed by performance on the Iowa Gambling Task. Developmental Psychology, 46(1), 193. 10.1037/a0016128 20053017

[R19] Chiu YC, & Lin CH (2007). Is deck C an advantageous deck in the Iowa Gambling Task? Behavioral and Brain Functions, 3, 1–11. 10.1186/1744-9081-3-37 17683599 PMC1995208

[R20] Chiu YC, Lin CH, Huang JT, Lin S, Lee PL, & Hsieh JC (2008). Immediate gain is long-term loss: Are there foresighted decision makers in the Iowa Gambling Task? Behavioral and Brain Functions, 4, 1–10. 10.1186/1744-9081-4-13 18353176 PMC2324107

[R21] Cohen J (1994). The earth is round (p < .05). American Psychologist, 49(12), 997–1003. 10.1037/0003-066X.49.12.997

[R22] de Visser L, van der Knaap LJ, van de Loo AJ, Van der Weerd CMM, Ohl F, & van den Bos R (2010). Trait anxiety affects decision-making differently in healthy men and women: Towards gender-specific endophenotypes of anxiety. Neuropsychologia, 48(6), 1598–1606. 10.1016/j.neuropsychologia.2010.01.027 20138896

[R23] Esfand SM, Null KE, Duda JM, de Leeuw J, & Pizzagalli DA (2024). Lifetime history of major depressive disorder is associated with decreased reward learning: Evidence from a novel online version of the probabilistic reward task. Journal of Affective Disorders, 350, 1007–1015. 10.1016/j.jad.2024.01.133 38278332

[R24] Eshel N, & Roiser JP (2010). Reward and punishment processing in depression. Biological Psychiatry, 68(2), 118–124. 10.1016/j.biopsych.2010.01.027 20303067

[R25] First MB, Williams JB, Karg RS, & Spitzer RL (2016). User’s guide for the SCID-5-CV Structured Clinical Interview for DSM-5^®^ disorders: Clinical version. American Psychiatric Publishing, Inc.

[R26] Funder DC, & Ozer DJ (2019). Evaluating effect size in psychological research: Sense and nonsense. Advances in Methods and Practices in Psychological Science, 2(2), 156–168. 10.1177/2515245919847202

[R27] Garrison J, Erdeniz B, & Done J (2013). Prediction error in reinforcement learning: A meta-analysis of neuroimaging studies. Neuroscience and Biobehavioral Reviews, 37(7), 1297–1310. 10.1016/j.neubiorev.2013.03.023 23567522

[R28] Gelman A, & Rubin DB (1992). Inference from iterative simulation using multiple sequences. Statistical Science, 7(4), 457–472. 10.1214/ss/1177011136

[R29] Grissom NM, & Reyes TM (2019). Let’s call the whole thing off: Evaluating gender and sex differences in executive function. Neuropsychopharmacology, 44(1), 86–96. 10.1038/s41386-018-0179-5 30143781 PMC6235899

[R30] Gupta R, Koscik TR, Bechara A, & Tranel D (2011). The amygdala and decision-making. Neuropsychologia, 49(4), 760–766. 10.1016/j.neuropsychologia.2010.09.029 20920513 PMC3032808

[R31] Haines N, Vassileva J, & Ahn W-Y (2018). The outcome-representation learning model: A novel reinforcement learning model of the Iowa Gambling Task. Cognitive Science, 42(8), 2534–2561. 10.1111/cogs.12688 30289167 PMC6286201

[R32] Hartung CM, & Lefler EK (2019). Sex and gender in psychopathology: DSM–5 and beyond. Psychological Bulletin, 145(4), 390–409. 10.1037/bul0000183 30640497

[R33] Haynes J, Haines N, Sullivan-Toole H, & Olino TM (2024).Test-retest reliability of the play-or-pass version of the Iowa Gambling Task. Cognitive, Affective, and Behavioral Neuroscience. 10.3758/s13415-024-01197-6 PMC11636993 38849641

[R34] Huang H, Thompson W, & Paulus MP (2017). Computational dysfunctions in anxiety: Failure to differentiate signal from noise. Biological Psychiatry, 82(6), 440–446. 10.1016/j.biopsych.2017.07.007 28838468 PMC5576575

[R35] Huggins AA, Weis CN, Parisi EA, Bennett KP, Miskovic V, & Larson CL (2021). Neural substrates of human fear generalization: A 7T-fMRI investigation. NeuroImage, 239, 118308. 10.1016/j.neuroimage.2021.118308 34175426

[R36] Huys QJ, Pizzagalli DA, Bogdan R, & Dayan P (2013). Mapping anhedonia onto reinforcement learning: A behavioural meta-analysis. Biology of Mood & Anxiety Disorders, 3(1), 12. 10.1186/2045-5380-3-12 23782813 PMC3701611

[R37] Hyde JS, Bigler RS, Joel D, Tate CC, & van Anders SM (2019). The future of sex and gender in psychology: Five challenges to the gender binary. American Psychologist, 74(2), 171. https://psycnet.apa.org/doi/10.1037/amp0000307 30024214 10.1037/amp0000307

[R38] Kenny DA (1995). The effect of nonindependence on significance testing in dyadic research. Personal Relationships, 2(1), 67–75. 10.1111/j.1475-6811.1995.tb00078.x

[R39] Li D, Wang J, & Ao M (2024). Numerical cognitive reflection, but not verbal cognitive reflection, moderates the association between trait anxiety and affective decision-making. Journal of Behavioral Decision Making, 37(1), e2359. 10.1002/bdm.2359

[R40] Li X, Lu Z-L, D’Argembeau A, Ng M, & Bechara A (2010). The Iowa Gambling Task in fMRI images. Human Brain Mapping, 31(3), 410–423. 10.1002/hbm.20875 19777556 PMC2826566

[R41] Mathews C (2001). Assortative mating in the affective disorders: A systematic review and meta-analysis. Comprehensive Psychiatry, 42(4), 257–262. 10.1053/comp.2001.24575 11458299

[R42] McGovern AR, Alexopoulos GS, Yuen GS, Morimoto SS, & Gunning-Dixon FM (2014). Reward-related decision making in older adults: Relationship to clinical presentation of depression. International Journal of Geriatric Psychiatry, 29(11), 1125–1131. 10.1002/gps.4200 25306937 PMC4353615

[R43] Moffitt TE, Arseneault L, Belsky D, Dickson N, Hancox RJ, Harrington H, Houts R, Poulton R, Roberts BW, & Ross S (2011). A gradient of childhood self-control predicts health, wealth, and public safety. Proceedings of the National Academy of Sciences, 108(7), 2693–2698. 10.1073/pnas.1010076108 PMC3041102 21262822

[R44] Morein-Zamir S, Shahper S, Fineberg NA, Eisele V, Eagle DM, Urcelay G, & Robbins TW (2018). Free operant observing in humans: A translational approach to compulsive certainty seeking. Quarterly Journal of Experimental Psychology, 71(10), 2052–2069. 10.1177/1747021817737727 PMC6159779 29359639

[R45] Mueller EM, Nguyen J, Ray WJ, & Borkovec TD (2010). Future-oriented decision-making in Generalized Anxiety Disorder is evident across different versions of the Iowa Gambling Task. Journal of Behavior Therapy and Experimental Psychiatry, 41(2), 165–171. 10.1016/j.jbtep.2009.12.002 20060098

[R46] Mukherjee D, & Kable JW (2014). Value-based decision making in mental illness: A meta-analysis. Clinical Psychological Science, 2(6), 767–782. 10.1177/2167702614531580

[R47] Must A, Horvath S, Nemeth VL, & Janka Z (2013). The Iowa Gambling Task in depression – what have we learned about suboptimal decision-making strategies? Frontiers in Psychology, 4. 10.3389/fpsyg.2013.00732 PMC3794198 24133474

[R48] Naugle RI, Chelune GJ, & Tucker GD (1993). Validity of the Kaufman brief intelligence test. Psychological Assessment, 5(2), 182. 10.1037/1040-3590.5.2.182

[R49] Nettle D, & Bateson M (2012). The evolutionary origins of mood and its disorders. Current Biology, 22(17), R712–R721. 10.1016/j.cub.2012.06.020 22975002

[R50] Nordsletten AE, Larsson H, Crowley JJ, Almqvist C, Lichtenstein P, & Mataix-Cols D (2016). Patterns of nonrandom mating within and across 11 major psychiatric disorders. JAMA Psychiatry, 73(4), 354–361. 10.1001/jamapsychiatry.2015.3192 26913486 PMC5082975

[R51] O’Doherty J, Kringelbach ML, Rolls ET, Hornak J, & Andrews C (2001). Abstract reward and punishment representations in the human orbitofrontal cortex. Nature Neuroscience, 4(1), 95–102. 10.1038/82959 11135651

[R52] Orsini CA, & Setlow B (2017). Sex differences in animal models of decision making. Journal of Neuroscience Research, 95(1–2), 260–269. 10.1002/jnr.23810 27870448 PMC5120608

[R53] Overman WH, & Pierce A (2013). Iowa Gambling Task with non-clinical participants: Effects of using real+ virtual cards and additional trials. Frontiers in Psychology, 4, 935. 10.3389/fpsyg.2013.00935 24376431 PMC3859904

[R54] Peters E, & Slovic P (2000). The springs of action: Affective and analytical information processing in choice. Personality and Social Psychology Bulletin, 26(12), 1465–1475. 10.1177/01461672002612002

[R55] Petry NM, Bickel WK, & Arnett M (1998). Shortened time horizons and insensitivity to future consequences in heroin addicts. Addiction, 93(5), 729–738. 10.1046/j.1360-0443.1998.9357298.x 9692271

[R56] Pike AC, & Robinson OJ (2022). Reinforcement Learning in Patients With Mood and Anxiety Disorders vs Control Individuals: A Systematic Review and Meta-analysis. JAMA Psychiatry, 79(4), 313. 10.1001/jamapsychiatry.2022.0051 35234834 PMC8892374

[R57] PVS Work Group. (2011). Positive Valence Systems: Workshop proceedings, National Institute of Mental Health. Retrieved from https://www.nimh.nih.gov/research/research-funded-by-nimh/rdoc/positive-valence-systems-workshop-proceedings

[R58] R Core Team (2022). R: A language and environment for statistical computing. R Foundation for Statistical Computing, Vienna, Austria. https://www.R-project.org/.

[R59] Sanislow CA, Pine DS, Quinn KJ, Kozak MJ, Garvey MA, Heinssen RK, ... & Cuthbert BN (2010). Developing constructs for psychopathology research: Research domain criteria. Journal of Abnormal Psychology, 119(4), 631. 10.1037/a0020909 20939653

[R60] Schneider W, Eschman A, Zuccolotto A, & Burgess S (2002). E-prime. Pittsburgh, PA: Psychology Software Tools, 19.

[R61] Siqueira A. S. S. de, Flaks MK, Biella MM, Mauer S, Borges MK, & Aprahamian I (2018). Decision making assessed by the Iowa Gambling Task and Major Depressive Disorder A systematic review. Dementia & Neuropsychologia, 12, 250–255. 10.1590/1980-57642018dn12-030005 30425788 PMC6200161

[R62] Smoski MJ, Lynch TR, Rosenthal MZ, Cheavens JS, Chapman AL, & Krishnan RR (2008). Decision-making and risk aversion among depressive adults. Journal of Behavior Therapy and Experimental Psychiatry, 39(4), 567–576. 10.1016/j.jbtep.2008.01.004 18342834 PMC2590786

[R63] Stan Development Team (2023a). Stan modeling language users guide and reference manual. Technical Report Version 2.32. http://mcstan.org

[R64] Stan Development Team (2023b). RStan: The R interface to Stan. R Package Version 2.26.22. http://mcstan.org

[R65] Steingroever H, Wetzels R, Horstmann A, Neumann J, & Wagenmakers E-J (2013a). Performance of healthy participants on the Iowa Gambling Task. Psychological Assessment, 25(1), 180–193. 10.1037/a0029929 22984804

[R66] Steingroever H, Wetzels R, & Wagenmakers E-J (2013b). A Comparison of Reinforcement Learning Models for the Iowa Gambling Task Using Parameter Space Partitioning. The Journal of Problem Solving, 5(2). 10.7771/1932-6246.1150

[R67] Sullivan-Toole H, Haines N, Dale K, & Olino TM (2022). Enhancing the psychometric properties of the Iowa Gambling Task using full generative modeling. Computational Psychiatry, 6(1), 189–212. 10.5334/2Fcpsy.89 37332395 PMC10275579

[R68] van den Bos R, Homberg J, & de Visser L (2013). A critical review of sex differences in decision-making tasks: Focus on the Iowa Gambling Task. Behavioural Brain Research, 238, 95–108. 10.1016/j.bbr.2012.10.002 23078950

[R69] van den Bos R, Jolles J, van der Knaap L, Baars A, & de Visser L (2012). Male and female Wistar rats differ in decision-making performance in a rodent version of the Iowa Gambling Task. Behavioural Brain Research, 234(2), 375–379. 10.1016/j.bbr.2012.07.015 22814113

[R70] Verdejo-Garcia A, Benbrook A, Funderburk F, David P, Cadet J-L, & Bolla KI (2007). The differential relationship between cocaine use and marijuana use on decision-making performance over repeat testing with the Iowa Gambling Task. Drug and Alcohol Dependence, 90(1), 2–11. 10.1016/j.drugalcdep.2007.02.004 17367959 PMC1986840

[R71] Wang T, Zeng J, Yuan Y, He Y, Zhu J, Lin B, Yin Q, & Peng P (2024). Exploring the complex relationship between depression and risky decision-making: A meta-analysis. Journal of Affective Disorders Reports, 100771. 10.1016/j.jadr.2024.100771

[R72] Werner NS, Duschek S, & Schandry R (2009). Relationships between affective states and decision-making. International Journal of Psychophysiology, 74(3), 259–265. 10.1016/j.ijpsycho.2009.09.010 19808059

[R73] Wetzels R, Vandekerckhove J, Tuerlinckx F, & Wagenmakers E-J (2010). Bayesian parameter estimation in the Expectancy Valence model of the Iowa gambling task. Journal of Mathematical Psychology, 54(1), 14–27. 10.1016/j.jmp.2008.12.001

[R74] Yerkes RM, & Dodson JD (1908). The relation of strength of stimulus to rapidity of habit-formation. Journal of Comparative Neurology and Psychology, 18, 458–482.

[R75] Zanini L, Picano C, & Spitoni GF (2024). The Iowa Gambling Task: Men and women perform differently. A meta-analysis. Neuropsychology Review, 1–21. 10.1007/s11065-024-09637-3 PMC11965174 38462590

[R76] Zhang F, Xiao L, & Gu R (2017). Does gender matter in the relationship between anxiety and decision-making? Frontiers in Psychology, 8, 281252. 10.3389/fpsyg.2017.02231 PMC5742200 29312077

[R77] Zhang L, Wang K, Zhu C, Yu F, & Chen X (2015). Trait anxiety has effect on decision making under ambiguity but not decision making under risk. PLoS ONE, 10(5), e0127189. 10.1371/journal.pone.0127189 26000629 PMC4441420

