# Supplementary material for: Reward and punishment learning among people with a lifetime history of anxiety, depression, and substance use disorder
Source: Cogn Affect Behav Neurosci. 2025 Jul 29;25(6):1765–78. doi: 10.3758/s13415-025-01331-y (PMC12615540; doi:10.3758/s13415-025-01331-y)
Supplement: Supplementary file 1 [file 13415_2025_1331_MOESM1_ESM.docx]

# Supplemental File 

## ORL Model Predictions

To illustrate the specific patterns of behavior captured by the ORL parameters, we conducted simulations wherein we manipulated the values of one parameter while holding the other parameters constant. We selected parameter values based on the posterior means of the person level parameters from the parent sample. Specifically, we used the highest and lowest person level parameter from the sample to illustrate differences within a parameter, and we used medians as “moderate” values to hold the other parameters constant.

Figure S1 shows the play proportions from 50 simulations of the ORL model. Based on the simulations, lower reward learning rates (*A*_rew_) are associated with playing less frequently on the bad decks because participants do not approach the higher-magnitude gains on those decks (see Table 3 in main text). Higher punishment learning rates (*A*_pun_) are also associated with playing less frequently on the bad decks (i.e., decks A & B) but, in contrast, because participants avoid the losses on those decks. This is particularly the case for deck B, which contains the largest single-trial loss (-$1150), and the model predicts that avoidance is greatest following the first encounter with that loss. Higher win frequency sensitivities (β*f*) are associated with playing more frequently on decks with more wins (decks B & D) and less frequently on decks with fewer wins and more losses (i.e., decks A & C). In particular, higher win frequency sensitivities are associated with playing less on deck A because this deck contains relatively few wins and the most losses compared to the other decks. Finally, higher response bias (β*b*) is associated with playing more frequently across all decks.

**Figure S1**

*ORL Model Predictions*

*Note.* Simulated choices from the ORL model at specific parameter values. Rows correspond to manipulations of the parameter according to a low (left column) and high (right column) values, while holding the other parameters at a moderate value.

## ORL-Self Report Correlations

We explored the relation between ORL parameters with self-reported measures of reward and punishment sensitivity from the Behavioral Inhibition/Behavioral Activation Scales (BIS/BAS; Carver & White, 1994). The BIS/BAS was used to assess reward (behavioral activation) and punishment (behavioral inhibition) sensitivity. The BIS provides a single score of avoidance behavior. The BAS provides a score of general approach behavior (BAS Total) as well as scores on subscales to provide specific measures of motivation to pursue goals (BAS Drive), desire and spontaneous approach towards rewards (cf. impulsive behavior; BAS Fun Seeking), and responsiveness to the anticipation and consumption of rewards (BAS Reward Sensitivity). To do this, we estimated correlations between BIS Total, BAS Total, BAS Drive, BAS Fun Seeking, and BAS Reward Sensitivity scores with the person-level estimates of the ORL parameters from each iteration of the posterior distributions. This generated distributions of correlations between BIS/BAS scores and ORL parameters, from which we calculated means and 95% CIs to obtain point estimates of the correlation (*r*) between the self-report measure and the ORL parameter, and to draw inferences based on the overlap of the 95% CI with 0.

Table S1 shows the correlations between the ORL parameters and scores on the BIS/BAS subscales. All correlations were weak; however, for multiple correlations, the 95% credible intervals (CIs) did not overlap with 0 (i.e., were ‘significant’). Specifically, higher punishment learning rates were associated with lower total BAS scores and lower scores on the BAS Drive and BAS Fun-Seeking subscales whereas higher punishment learning rates were associated with higher BIS Total scores. Higher win frequency sensitivities were associated with lower scores on the BAS Total, BAS Drive, and BAS Fun Seeking subscales whereas higher win frequency sensitivities were associated with higher BIS Total scores. Finally, higher estimates of bias were associated with higher BIS Total scores. No associations between BIS/BAS scores were found with reward learning rates. Thus, self-reported measures of reward and punishment sensitivity were associated with several measures of IGT task performance.

Despite the low strength of these relations, the pattern of findings between the self-report and ORL parameters are consistent with those presented in the main text. Specifically, the behavioral inhibition system, measured by the BIS Total, is heightened in anxiety (Carver & White, 1994) and here we show that a self-reported measure of heightened BIS is associated with higher punishment learning rates. In addition, the behavioral activation system, measured by the BAS scale and subscales, is diminished in depression and here we show that self-reported measures of diminished BAS are associated with higher win frequency sensitivities. Although not significant, we also found elevated win frequency sensitivities among those diagnosed with depression (see Figure 2 in the main text). Thus, our results suggest some convergent findings between ORL parameters with diagnostic and self-reported measures related to anxiety and depression.

| **Table S1** *Correlations Between ORL Model Parameters & BIS/BAS Scores* |  |  |  |  |
| --- | --- | --- | --- | --- |
|  | ORL Parameters |  |  |  |
| Self-Report | *A+* | *A-* | *βf* | *βb* |
| BAS Total | .07 [-.06,.18] | **-.15 [-.22,-.09]** | **-.07 [-.14,-.01]** | .02 [-.05,.09] |
| BAS Drive | .06 [-.06,.17] | **-.16 [-.22,-.09]** | **-.11 [-.18,-.05]** | .00 [-.07,.07] |
| BAS Fun Seeking | .05 [-.07,.17] | **-.10 [-.17,-.04]** | **-.08 [-.14,-.01]** | .06 [-.01,.13] |
| BAS Reward Responsivity | .05 [-.06,.17] | **-.11 [-.18,-.05]** | .01 [-.05,.08] | .00 [-.07,.07] |
| BIS Total | .03 [-.09,.15] | **.11 [.03,.18]** | **.07 [0,.14]** | **.11 [.04,.19]** |
| *Note.* Bolding represents correlations in which 95% CIs do *not* overlap with 0. |  |  |  |  |

## IGT Performance & Anxiety

***Presence vs. Absence Differences***

	In the main text, we show that punishment learning rates are higher among those with a history of anxiety than those without a history of anxiety. According to the model, higher punishment learning rates should be associated with avoidance (i.e., passing) following losses. As shown above (Figure S1), higher punishment learning rates capture greater avoidance of the bad decks, particularly deck B. Our data are largely consistent with this finding. In the top row of Figure S2, we overlay observed and model-predicted play proportions across trials for deck B at the group-level (left panel) and for two participants (right panel), one with and one without anxiety. Punishment learning rates for these two participants were closest to their respective group-level mean punishment learning rates. Overall, participants with anxiety were less persistent in choosing deck B than those without anxiety.

	In the Discussion, we note that participants with a history of anxiety may have chosen deck B to avoid the uncertain losses associated with that deck, assuming that the infrequent losses in deck B function similar to uncertain losses. If anxiety is characterized by specific avoidance of uncertain losses in the IGT, we would also expect participants with anxiety to avoid deck D because this deck has the same frequency of losses as deck B, but we did not find this. To illustrate, the bottom row of Figure S2 shows trial-level play proportions for deck D among those with and without a history of anxiety. There is clearly not a pattern in which individuals with anxiety avoid this deck. Instead, there is a tendency in which participants with a history of anxiety played on deck D more frequently than participants without a history of anxiety.

**Figure S2**

*Play Proportions in Anxiety*

*Note.* Observed (datapoints) and model-predicted (datapaths) play proportions across trials for decks B and D among individuals with (Anx+) and without a lifetime history of anxiety (Anx-). Error bands represent 50 % (darker) and 95% (lighter) credible intervals from the ORL.

Figure S2 shows that although participants with a history of anxiety tended to avoid deck B more than those without that history, this difference was small. Elevated punishment learning rates should be associated with more avoidance behaviors following a loss; that is, passing should be more likely on the trials immediately after receiving a loss. As the simulations show in Figure S1, this avoidance should be greatest for deck B, particularly after the first loss on that deck. To probe this, we calculated the proportion of participants who passed during the first 10 trials following the first loss on each deck. These data are shown in Figure S3. With the exception of deck A, participants with anxiety were more likely to pass immediately (i.e., during the first trial) following the first loss on each deck. For deck B, this difference lingered such that participants with anxiety passed more frequently during the 10 trials following the first loss. Although the differences in passing between those with and without anxiety were small (note the *y* axes), these differences align with what we would predict with elevated punishment learning rates in anxiety. 

**Figure S3**

*Avoidance Behavior in Anxiety*

*Note.* Proportion of participants with and without anxiety who passed following the first loss on the IGT during the first 10 trials after that loss.

***Current vs. Past Differences***

Given that we had a larger number of individuals with current (*n* = 65) and past (*n* = 28) history of anxiety, we examined whether parameters differed as a function of current or remitted anxiety. To do this, we fit the ORL model to those with a history of anxiety and included a beta coefficient to compare those with current and remitted anxiety. Overall, there were no differences between those with a current or past history of anxiety, illustrated in Figure S4. The data and code for this analysis are available at [https://osf.io/fg7sj/?view_only=cb5b3f32171c4309bf5438784c2d2e98](https://osf.io/fg7sj/?view_only=cb5b3f32171c4309bf5438784c2d2e98).

**Figure S4**

*Posterior Distributions for Current vs. Past History of Anxiety*

*Note.* Posterior distributions of reward learning rates (*A+*), punishment learning rates (*A-*), win frequency sensitivities (*βf*), and bias parameters (*βb*) from the PP-ORL model fit to those with a history of anxiety only. The left column shows group-level means for each parameter (rows) across those with current and past anxiety. The right column shows mean-differences between current and past history of anxiety, where a higher mean-difference corresponds to elevated parameter values among those with current anxiety. Data points with lines above each distribution represent posterior means ±95% credible intervals.

## *Non-Independence from Assortative Mating*

	Because the sample consisted of mother-father dyads, some of the IGT data included both mothers and fathers within the same dyad and thus could result in non-independence that could bias statistical inferences (Kenny, 1995). This is particularly a concern for the finding that punishment learning rates were elevated among those with anxiety as assortative mating, when individuals mate with others sharing similar phenotypes, has been shown with affective disorders, including anxiety (Mathews, 2001; Nordsletten et al., 2016). To probe this issue, we examined correlations between punishment learning rates among mothers and fathers who were both included in the sample. This correlation was positive, but weak (*r* = .12), indicating dependence may not be particularly problematic in our data. In spite of this, however, we conducted a sensitivity analysis in which we removed one participant from each dyad and reran our analyses. This analysis replicated our main finding that punishment learning rates are elevated among those with anxiety (posterior mean *β*_*Anx*_ = 0.17, 95% CI [0.03,0.31]). The code for this analysis is available at [https://osf.io/fg7sj/?view_only=cb5b3f32171c4309bf5438784c2d2e98](https://osf.io/fg7sj/?view_only=cb5b3f32171c4309bf5438784c2d2e98).

## Sex-Differences in ORL Parameters

	Our results showed that female participants had lower punishment learning rates and lower response bias than male participants. This finding characterized a pattern of behavior in which male participants showed greater separation in playing on the good decks over the bad decks than female participants (see Figure 3 in the main text). To further illustrate these differences, Figure S5 shows violin plots of the observed session-wide play proportions, constructed from individual participant data. These plots also show observed (horizontal bars) and model-predicted (open datapoints) group-level means for male and female participants. Overall, female participants showed a trend towards playing on the good decks less frequently and on the bad decks more frequently than male participants, a finding that is consistent with the literature (Zanini et al., 2024).

**Figure S5**

*Session-Wide Play Proportions for Male & Female Participants*

*Note.* Violin plots of the session-wide play proportions for each deck, constructed from individual participant data, split by male and female participants. Observed (horizontal bars) and model-predicted (open datapoints) group-level mean play proportions are also depicted for male and female participants.

## Parameter Recovery

***ORL Model Parameter Recovery***

	We examined whether parameters from the ORL model could be recovered to diagnose estimation issues. To do this, we simulated data for 293 participants using the same diagnostic-assignments as in the data (i.e., 32% with anxiety, 31% with depression, & 29% with substance use disorder). Person-level parameters for each participant were simulated using the parameters from participants’ corresponding group-level mean and the group-level standard deviations. For example, the simulated *βf* parameter (i.e., win frequency sensitivity) for each participant was obtained by drawing from the following distribution:

|  | $βf_{i} \sim Normal(3.11+0.00X_{Anx, i}+0.94X_{Dep, i}+0.09X_{SUD, i}-0.06X_{Sex, i}, 3.59).$ |  |
| --- | --- | --- |

where the mean of the normal distribution (i.e., *μ*) is equivalent to Equation 9 from the main text but with the posterior means of the *β* parameters from the model represented within the equation. The standard deviation of the normal distribution (i.e., *σ*) was the posterior mean of the group-level standard deviation from the model. After simulating parameters for each participant, we generated choice data for each participant and then fit the ORL model to the generated data to determine whether the simulated parameters could be recovered.

	Overall, parameters showed adequate recovery. Figure S3 shows correlations between simulated and recovered person-level parameters. This plot shows posterior means of the recovered parameters as a function of the simulated (labeled *actual*) parameters. The correlations between recovered and actual parameters were positive and strong, although reward learning rates (*A*-) showed weaker recovery than the other parameters. To probe why reward learning rates may not have recovered as well, we examined collinearity between the recovered parameters. Reward learning rates showed a moderately positive correlation with response bias (β*b*), *r* = .33. This could explain the weaker recovery of the reward learning rates, indicating some degree of tradeoff between estimates of reward learning rates and response bias parameter.

**Figure S3**

*Parameter Recovery for ORL Model*

*Note.* Recovered parameters as a function of the actual parameters from the ORL model. Parameters were *z*-scored for visualization purposes.

The posterior distributions of the recovered *μ* and *β* parameters are displayed in Figure S4 (diagnostic group-comparisons) and Figure S5 (sex-comparisons). These posterior distributions capture the main findings that the presence of anxiety was associated with higher punishment learning rates (posterior mean *β* = .15, 95% CI [.01, .29]), and female participants had lower punishment learning rates (posterior mean *β* = -.18, 95% CI [-.33, -.04]) and lower response bias (posterior mean *β* = -.24, 95% CI [-.48, .01]) than male participants. Thus, recovered parameters largely reflect the same findings as those presented in the main text.

**Figure S4**

*Posterior Distributions of Recovered Parameters for Diagnostic Group-Comparisons*

*Note.* Posterior distributions of the recovered reward learning rates (*A+*),  punishment learning rates (*A-*), win frequency sensitivities (*βf*), and bias parameters (*βb*) from the PP-ORL model, fit to the simulated data to assess parameter recovery. The left column shows group-level means for each recovered parameter (rows) across anxiety (Anx), depression (Dep), substance use disorder (SUD), and healthy controls (HC). The right column shows mean-differences between the presence and absence of anxiety, depression, and substance use disorder for each recovered parameter. Data points with lines above each distribution represent posterior means ±95% credible intervals.

**Figure S5**

*Posterior Distributions of Recovered Parameters for Male & Female Participants*

*Note.* Posterior distributions of the recovered reward learning rates (*A+*),  punishment learning rates (*A-*), win frequency sensitivities (*βf*), and bias parameters (*βb*) from the PP-ORL model, fit to the simulated data to assess parameter recovery. The left column shows group-level means for each recovered parameter (rows) across male and female participants. The right column shows mean-differences between male and female participants, where a higher mean-difference corresponds to elevated parameter values among female participants. Data points with lines above each distribution represent posterior means ±95% credible intervals.
